# Dynamics Evolution of Flavor and Quality Attributes in Three-Cup Chicken: Insights from Multi-Technical Analysis During Stewing

**DOI:** 10.3390/foods14223970

**Published:** 2025-11-19

**Authors:** Qianzhu E, Yuting Wang, Yuwei Liu, You Long, Chang Li, Jianhua Xie, Qiang Yu, Yi Chen

**Affiliations:** State Key Laboratory of Food Science and Resource, Nanchang University, Nanchang 330047, China; ncuspyeqianzhu@163.com (Q.E.); wangyuting@ncu.edu.cn (Y.W.); 417900210143@email.ncu.edu.cn (Y.L.); longyou1016@163.com (Y.L.); lichang@ncu.edu.cn (C.L.); jhxie@ncu.edu.cn (J.X.); yuqiang8612@163.com (Q.Y.)

**Keywords:** flavoromics, three-cup chicken, salty and umami, key flavor compounds, GC-MS, GC-IMS

## Abstract

Three-Cup Chicken, a traditional Hakka dish, is known for its distinctive umami and salty flavor profile. However, the dynamic evolution of key flavor compounds and associated physicochemical attributes during its characteristic stewing process remains inadequately characterized. Therefore, this study investigated flavor and quality changes in Three-Cup Chicken during stewing using an integrated analytical approach, including gas chromatography-mass spectrometry (GC-MS), gas chromatography-ion mobility spectrometry (GC-IMS), E-tongue, and E-nose, alongside analyses of texture, color, pH, total volatile basic nitrogen (TVB-N), thiobarbituric acid-reactive substances (TBARS), and moisture content. The results revealed that prolonged stewing promoted lipid oxidation, increased hardness, enhanced redness and yellowness, while moisture content gradually decreased. Electronic tongue and nose analyses revealed an increase in saltiness, umami, and sulfur compounds during stewing, complemented by a significant rise in umami amino acids from further analysis. Ten important taste compounds with variable importance in projection (VIP) > 1 and odour activity value (OAV) > 1 were filtered out of 137 volatile compounds, the majority of which were aldehydes. These research findings clearly demonstrate the formation and evolution patterns of the savory and salty flavor profile in Three-Cup Chicken, offering theoretical underpinnings as well as helpful advice for maximizing the dish’s genuine flavor.

## 1. Introduction

Traditional cuisine constitutes an important component of global culinary cultural heritage, with its unique flavors serving as a distinct expression of cultural identity. However, industrialization and standardization pose significant challenges to the authentic preservation and quality control of these traditional flavors. In China, traditional cuisine exhibits remarkable regional diversity, among which Hakka cuisine is particularly renowned for its distinctive savory and umami characteristics. A representative example is Three-Cup Chicken, a classic dish known for its rich aroma and balanced salty and umami profile. Elucidating the flavor formation mechanisms in such dishes is crucial not only for advancing flavor science but also for culinary standardization, quality assurance, and cultural heritage safeguarding.

Named after the traditional recipe that uses a cup each of rice wine, soy sauce, and tea oil with no additional water when stewing chicken, Three-Cup Chicken is a well-known home-style dish with origins in Ganzhou City, Jiangxi Province, China [1]. It is an integral part of both Gan and Hakka culinary traditions. The dish is characterized by its tender and chewy meat, robust aroma, and a harmonious blend of sweetness, saltiness, and umami.

One of the most important dietary senses is flavor, particularly odor. Even though volatile chemicals in chicken flesh and broth have been the subject of countless studies [2], such as Qingyuan chicken [3], Zanthoxylum bungeanum essential oil chicken soup [4], Bian chicken [5], but the technique by which Three-Cup Chicken produces volatile organic compounds (VOCs) while it is stewing is still not fully understood. At the same time, growing consumer demand for consistently high-quality processed foods underscores the necessity of investigating quality changes and key flavor compounds throughout the cooking process. Analyzing VOCs and taste variations in Three-Cup Chicken is essential for optimizing its overall flavor profile.

The flavor of cooked chicken is complex, whereas the smell of raw chicken is nearly nonexistent and bloody. The meat’s distinctive aroma is created when non-volatile components in the muscle and fat tissues react during cooking to release a significant amount of volatile chemicals [6]. The *Maillard* reaction, Strecker degradation, lipid oxidation, lipid-Maillard interaction, and thiamine degradation are the key mechanisms for the creation of taste components [7]. Yang et al. [8] found that stewing is a crucial step in the Wuding chicken process that creates flavorful and fresh flavors. According to Wang et al. [9], heating decreased the amount of certain amino acids, carbohydrates, and nucleic acids in chicken meat, and these substances became partially soluble in the broth. Therefore, the quality and flavor development of three-cup chicken are greatly influenced by the braising process. Pu et al. [10] find that longer stewing times led to a progressive loss of elasticity and cohesiveness, a notable drop in the moisture content and endogenous fluorescence intensity of the protein in braised beef, a notable rise in the amount of TBARS and protein carbonyl groups, and an increase in the hardness and chewiness of the braised beef.

The headspace solid-phase microextraction combined with gas chromatography-mass spectrometry (HS-SPME-GC-MS), gas chromatography-olfactometer coupling (GC-O), electronic nose, and electronic nose, and gas chromatography-ion mobility spectrometry (GC-IMS) are currently the primary techniques for studying volatile flavors in meat products [11]. The advantages of great sensitivity, strong selectivity, and objective correctness allow all of these methods to be combined to produce more comprehensive, reliable, and scientific information regarding food flavor [12]. By using the double-jointed wings of ducks as a model, based on HS-SPME-GC-MS and HS-GC-IMS, Wang et al. [13] examined the effects of various stewing cycles on traditional Chinese stewed soup. Their findings demonstrated that, after 15 cycles of stewing, the soup’s flavor and aroma components were progressively enhanced and stabilized. Using HS-GC-IMS, high-performance liquid chromatography (HPLC), E-nose, and E-tongue, Liu et al. [14] examined how several process steps affected the volatile taste and its precursors during the manufacturing of beer fish. Yao et al. [15] used the HS-SPME-GC-MS, HS-GC-IMS, and electronic nose techniques to analyze the taste profiles of roasted lamb seasoned with salt, cumin, and chile. Research on the volatile flavorings of three-cup chicken is lacking, nevertheless.

However, despite these advancements and the established understanding of general meat flavor chemistry, a significant research gap remains concerning the volatile flavor profile of Three-Cup Chicken. Previous studies have investigated flavor formation in various chicken dishes such as Qingyuan chicken [3], Zanthoxylum bungeanum essential oil chicken soup [4], and Bian chicken [5]. However, the dynamic evolution of flavor compounds throughout the characteristic stewing process of Three-Cup Chicken remains unexplored. Specifically, the systematic correlation between key physicochemical properties (e.g., texture, color, pH, thiobarbituric acid-reactive substances (TBARS), total volatile basic nitrogen (TVB-N)), sensory attributes (e.g., saltiness, umami), and the precise volatile compound formation during stewing has not been thoroughly investigated for this traditional dish. This study provides the first comprehensive investigation into the dynamic evolution of flavor and quality attributes in Three-Cup Chicken during its characteristic stewing process. The originality of this research lies in its integrated multi-technical approach that simultaneously tracks the complex interplay between volatile aroma compounds, taste profiles, and physicochemical transformations throughout the cooking progression. This study is expected to elucidate the dynamic evolution of characteristic flavor and quality attributes of Three-Cup Chicken, providing a scientific foundation for its standardized processing and industrial production. Such efforts will deepen understanding of traditional food flavor chemistry and accelerate its industrial translation.

## 2. Materials and Methods

### 2.1. Raw Materials and Chemicals

Chicken, green onion, ginger, rice wine (Shenlin brand, Hubei Shenlin Liquor Industry Co., Ltd., Xiaogan, China), soy sauce (Chubang brand, Zhongshan Chubang Seasoning Food Co., Ltd., Zhongshan, China), and tea oil (Qiyunshan brand, Jiangxi Qiyunshan Native Produce Co., Ltd., Ganzhou, China) were purchased from Tianhong Shopping Center, Jiangda South Road, Nanchang, Jiangxi Province, China. The chicken was transported to the laboratory under refrigeration (4 °C) within 1 h after purchase. The meat was obtained from chickens slaughtered within 24 h post-mortem, with an initial pH of 5.9 ± 0.1 (measured using a portable pH meter), and was used immediately for sample preparation.

Boric acid and magnesium oxide were purchased from Xilong Science Co., Ltd. (Shantou, China); tartaric acid was purchased from Yuanye Biotechnology Co., Ltd. (Shanghai, China); trichloroacetic acid solution, 50% (*w*/*v*) was purchased from Shanghai McLean Biochemistry and Technology Co. (Shanghai, China), Shanghai Aladdin Reagent Co., Ltd. (Shanghai, China); 2-methyl-3-hexanone (chromatographic grade) and n-alkanes (C_8_–C_24_) were purchased from Sigma-Aldrich Company, St. Louis, MO, USA. Other chemicals and reagents utilized in the study were of analytical grade.

### 2.2. Preparation of Three-Cup Chicken Samples with Different Stewing Times

The stewing time intervals (4, 8, 12, 16, and 20 min) were selected based on preliminary experiments that monitored the key physicochemical parameters—color, texture, and GC-IMS—which are direct outcomes of the *Maillard* reaction and protein denaturation. This range was confirmed to cover the critical phases of sensory development, with the upper limit also informed by traditional culinary practice.

A precise mixture of 500.00 g of chicken cubes (2 × 2 × 2 cm^3^), 50.00 g of rice wine, 50.00 g of soy sauce, 50.00 g of tea oil, 20.00 g of green onion, and 20.00 g of ginger was placed into a 3.2 L Bear earthenware casserole (Model: CP-G0047; top inner diameter: 19 cm). No additional water or broth was added, as the moisture released from the chicken and the added sauces constituted the cooking medium. The cooking process using a Bear electric ceramic cooker (Model: DTL-A22D1) at a constant power of 1600 W. No additional liquid was added. The pot was kept covered during the entire stewing process. The timing for each interval was strictly controlled using a laboratory timer. Immediately upon reaching each time interval, the casserole was removed from the heat source and uncovered to terminate the cooking process.

### 2.3. Basic Physical and Chemical Indicators of Three-Cup Chicken

#### 2.3.1. TVB-N Measurement

The determination was carried out according to the automatic Kjeldahl method in the Chinese standard (GB 5009.228–2016 [16]). Weigh the appropriate amount of sample in a digestion tube, add 50 mL of water to disperse evenly, add 1 g of magnesium oxide, immediately connected to a distiller, using Kjeldahl nitrogen determination (Jinan Haineng Instrument Co., Ltd., Jinan, China, K9840) to determine, and at the same time do the reagent blank.

#### 2.3.2. pH Measurement

Chicken meat was ground and weighed 5.00 g, mixed with 50.0 mL of KCl solution, homogenized for 60 s, and then left to stand for 35 min before being tested using a pH meter.

#### 2.3.3. TBARS Measurement

The TBARS value of chicken meat was determined with reference to Chinese standard (GB 5009.181-2016 [17]). The chicken meat was minced and weighed 4.00 g in a 50 mL centrifuge tube, 20 mL of 7.5% trichloroacetic acid (TCA) solution was added, vortexed, and shaken for 60 s. The solution was stirred well, and then centrifuged at 4800× *g* for 15 min after 30 min of standing at room temperature. 4 mL of the supernatant was then pipetted into a test tube, 4 mL of 0.02 mol/L thiobarbituric acid solution was added, and heated at 90 °C in a water bath for 30 min, followed by heating. The reaction solution was heated in a water bath at 90 °C for 30 min, then removed and immediately placed in an ice-water bath for 10 min, and the absorbance was measured at 532 nm and 600 nm, respectively. The results were expressed as malondialdehyde (MDA) (mg MDA/kg sample).

#### 2.3.4. Color Measurement

Randomly selected chicken pieces were measured for color. A colorimeter (Colorimeter II Reflectance, Minolta, Osaka, Japan) was used for determining the chromaticity of the chicken meat studied by applying the head of the colorimeter directly to the sample surface. Results are shown as L* (luminance, from 0 = black to 100 = white), a* (>0 = red, <0 = green) and b* (>0 = yellow, <0 = blue).

#### 2.3.5. Textural Profile Analysis

A texture analyzer (TA.XTPlus, Texture Technologies, Hamilton, MA, USA) was used to examine the textural characteristics of heated meat cubes (2 × 2 × 2 cm^3^). A P/36R cylinder probe was used for a double compression cycle test. 30% compression, a test speed of 1 mm/s, a pre-test speed of 2 mm/s, and a post-test speed of 2 mm/s were the test settings.

The chicken meat was minced and weighed 4.00 g in a 50 mL centrifuge tube, 20 mL of 7.5% trichloroacetic acid (TCA) solution was added, vortexed, and shaken for 60 s. The solution was stirred well, and then centrifuged at 4800× *g* r/min for 15 min after 30 min of standing at room temperature. 4 mL of the supernatant was then pipetted into a test tube, 4 mL of 0.02 mol/L thiobarbituric acid solution was added, and heated at 90 °C in a water bath for 30 min, followed by heating. The reaction solution was heated in a water bath at 90 °C for 30 min, then removed and immediately placed in an ice-water bath for 10 min, and the absorbance was measured at 532 nm and 600 nm, respectively. The results were expressed as malondialdehyde (MDA) (mg MDA/kg sample).

### 2.4. Water Analysis

The direct drying method was used to determine the chicken’s water content. The DHG-9246A Electric Blast Drying Oven (Jinghong Test Equipment Co., Ltd., Shanghai, China) was used to dry about 2.00 g of roasted chicken samples at 105 °C until their weight remained constant. Using an NMI20-NMR analyzer (Niumag Co., Ltd., Shanghai, China), the distribution of water was ascertained. The Carr–Purcell–Meiboom–Gill (CPMG) sequence was used to determine the spin–spin relaxation time (T2), and the pertinent parameters were adjusted slightly from Wang et al. [18]: TW = 3500 ms; NECH = 4000; NS = 8.

### 2.5. Flavor Change Analysis

#### 2.5.1. Determination of Electronic Nose

Weigh out 2.00 g of minced chicken breast, place it in an electronic nose sample bottle, then screw on the lid. Set the electronic nose parameters as follows: detection time 180 s, cleaning time 150 s, and gas flow rate 2.5 mL/min. The detection was carried out using the portable E-nose device PEN3 from Germany which are consists of ten sensors, the standard sensor performance of the PEN3 electronic nose is shown in Table S1 [19].

#### 2.5.2. Determination of Electronic Tongue

Place 5.00 g of stranded samples in a centrifuge tube, fill it with 25 mL of ultrapure water, homogenize for 30 s, centrifuge at 11,000× *g* for 10 min at 4 °C, let it stand, remove the supernatant, filter it, expand it to 80 mL for measurement, and perform three parallels for every set of samples.

#### 2.5.3. Determination of Free Amino Acid (FAA) Content

A centrifuge tube was filled with 1.00 g of pulverized sample, 5 mL of 8% sulfosalicylic acid solution, homogenized for 30 s, and then centrifuged at 11,000× *g* for 10 min at 4 °C. Following centrifugation, the sample was left to stand for 10 min. 2 mL of the supernatant were then combined with 2 mL of hexane and centrifuge at 11,000× *g* for 10 min at 4 °C. After filtering the lower supernatant over a 0.2 μm membrane, the amount of free amino acids in the sample was measured using an automated amino acid detector [20].

The amino acid composition was used to calculate the following taste-related amino acid indices, based on established literature [20]: Uaa (Umami Amino Acids): The sum of the concentrations of umami-tasting amino acids, specifically Aspartic acid (Asp) and Glutamic acid (Glu). Saa (Sweet Amino Acids): The sum of the concentrations of sweet-tasting amino acids, including Glycine (Gly), Alanine (Ala), Serine (Ser), Threonine (Thr), and Proline (Pro). Baa (Bitter Amino Acids): The sum of the concentrations of bitter-tasting amino acids, including Valine (Val), Leucine (Leu), Isoleucine (Ile), Phenylalanine (Phe), Tyrosine (Tyr), Lysine (Lys), Methionine (Met), and Histidine (His).

#### 2.5.4. Determination of Volatile Substances (GC-IMS)

A sample (1.00 g) was introduced into a 20 mL headspace vial for analysis.

GC conditions: the HP-5 column (15 m × 0.53 mm) was maintained at a constant temperature of 80 °C; the carrier gas was N_2_; the injection settings for the headspace were 500 μL and 15 min of incubation at 80 °C, the initial flow rate was 2 mL/min, and the flow rate was held for 2 min and then increased to 20 mL/min at 2.25 mL/min, rising at 10 mL/min to 150 mL/min.

The analysis of the n-ketone (C_4_–C_9_) mixture resulted in linear curves for the retention time and retention index. Using the VOCal program, the GC retention index database and the IMS migration time database were searched and compared to determine the retention indices of the compounds.

#### 2.5.5. Determination of Volatile Substances (HS-SPME-GC-MS)

SPME conditions: A sample of chicken breast was taken, broken, and mixed with a homogenizer. Accurately weigh 2.00 g, put it in a 20 mL headspace bottle, add 10.0 μL of 2-methyl-3-heptanone internal standard (dissolved in methanol) with a concentration of 40 μg/mL and 2 mL of saturated NaCl solution, sealed with a lid, then insert the 65 μm CAR/PDMS extraction head, equilibrate at 80 °C under a water bath for 8 min, adsorb it for 40 min, take it out, remove it, and then insert it into the GC After removal, it was inserted into the GC inlet and desorbed at 250 °C for 5 min to start the instrument to collect data.

GC conditions: HP-5 MS column (30 m × 250 μm, 0.25 μm); carrier gas (He) flow 1.2 mL/min inlet temperature of 250 °C; the initial temperature of 40 °C, held for 2 min; 5 °C/min rose to 90 °C, held for 3 min; then 8 °C/min rose to 170 °C, held for 3 min; finally 10 °C/min rose to 250 °C/min to 250 °C and kept for 2 min.

MS conditions: electron energy 70 eV, mass stroke m/z 30~500, detector temperature 150 °C, transmission line temperature 280 °C, and electron ion source temperature 230 °C.

Volatiles were identified by comparing the mass spectra of all detected compounds with the mass spectra from the NIST 20 library. Results were accepted when both the match and the inverse match were above 700.

### 2.6. Statistical Analysis

All data were processed and visualized using Origin 2021 (OriginLab, Northampton, MA, USA) and TBtools-II software(TBtools-II (Toolbox for Biologists) v2.322). Statistical analysis was performed using SPSS Statistics 25.0 (IBM Corp., Armonk, NY, USA). Significant differences among chicken samples were analyzed by one-way analysis of variance (ANOVA) followed by Duncan’s test at a significance level of 0.05. All experiments were conducted with three independent replicates, and results are expressed as mean ± standard error. Multivariate statistical analysis, including principal component analysis (PCA) and variable importance in projection (VIP) analysis, was performed using SIMCA 14.1 (Umetrics Co., Ltd., Umea, Sweden).

## 3. Results and Discussion

### 3.1. The Analysis of Oxidation Indicators

**Figure 1:**
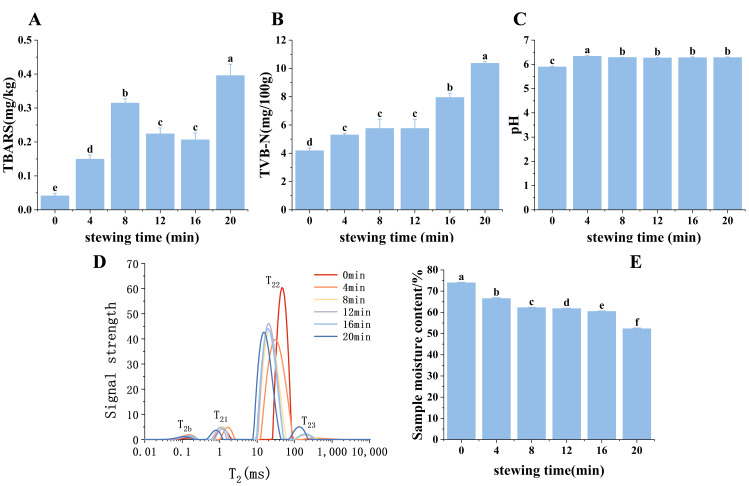
Oxidation indicators and moisture changes during the stewing process of three-cup chicken (**A**) TBARS, (**B**) TVB-N, (**C**) pH value, (**D**) T_2_ relaxation time distribution curve, (**E**) moisture content changes. Different letters indicate significant differences among different treatments (*p* < 0.05).

TBARS value is an indicator for the concentration of malonaldehydes (MDA), which are secondary products of lipid oxidation [21]. The changes in TBARS values reflect the extent of lipid oxidation in the samples during stewing. A higher TBARS value indicates more oxidation, while a lower TBARS value indicates less oxidation. As illustrated in Figure 1A, the TBARS values showed a fluctuating trend with increasing cooking time, initially increasing, then decreasing between 8–12 min, before increasing again (*p* < 0.05). The observed decrease after 8 min may be attributed to the breakdown and subsequent reactions of malondialdehyde (MDA). As in fried and grilled fish fillets, the final MDA formed may be lost due to dissolution in frying oil or due to adduct formation with proteins and possibly interaction with other components of the fish [22]. High temperatures not only induce an increase in TBARS values but also prompt them to react further with other components, also leading to a decrease in TBARS values [23]. The mechanisms responsible for this phenomenon remain to be further explored [24]. The TBARS value reached its highest level (0.40 mg MDA/kg) after 20 min of stewing. According to Wu et al. [25], off-flavors typically develop when TBARS values exceed 1.0 mg MDA/kg, which is considered the beginning of sensory perception of lipid oxidation. All TBARS values measured in this study remained well below this threshold, indicating that lipid oxidation was effectively controlled throughout the stewing process.

TVB-N value, which refers to the enzymes and microorganisms that cause the proteins in the meat to break down to produce ammonia, amines, and other volatile, toxic alkaline nitrogenous substances, is the primary indicator of freshness, or the degree of spoilage of animal food. The higher the value, the more spoiled the meat is [26]. As illustrated in Figure 1B, the volatile amino nitrogen concentration of the chicken meat gradually increased as the stewing time was prolonged. This implies that the level of protein oxidation in the chicken also grew progressively. The TVB-N value reached its maximum (10.36 mg/100 g) at 20 min of stewing. This value remains substantially below the spoilage threshold of >25 mg/100 g generally accepted for meat products [27]. The results demonstrate that the stewing process, even at the maximum duration tested, did not induce protein degradation to levels associated with spoilage.

As shown in Figure 1C, when compared to raw meat, the pH of all groups increased after stewing (*p* < 0.05). This phenomenon could be attributed to heat-induced denaturation of myofibrillar proteins, which alters their tertiary and quaternary structures, thus reducing or encapsulating acidic groups and raising the pH.The three-cup chicken meat’s pH steadily dropped as the stewing time increased. This could be because the proteins’ chemical bonds and spatial organization were further broken, leading to a sequence of reactions like protein denaturation, degradation, and oxidation that lowered the meat’s pH [28].

### 3.2. The Analysis of Texture and Chromaticity

From Table 1, heating significantly affected the hardness, gumminess, and chewi-ness of chicken, and several parameters showed similar trends during the cooking pro-cess: with the prolongation of stewing, the hardness and gumminess of the three cups of chicken increased gradually, the springiness and chewiness appeared to increase and then decrease (*p* < 0.05), and there were marginal changes in cohesiveness, and the resili-ence gradually decreased. The texture of the meat is affected by the moisture content in the meat, myofibrillar proteins, collagen, elastin and myofibrils own properties and in-teractions, heating causes myofibrillar proteins protein denaturation contraction and coagulation, and thermal denaturation of collagen also causes contraction of myofibrils, which results in tightening of the chicken meat, causing an increase in the hardness of the chicken meat [29]. The increase in the elasticity of chicken meat may be due to the gradual dissolution of thermally soluble collagen to form gelatin on the one hand, and myofibrillar proteins absorbing water and swelling on the other hand.

Table 1 shows that cooking has a considerable impact on the L* value of chicken meat. Compared to the raw chicken flesh, the 4-min cooked sample’s L* value was considerably greater (*p* < 0.05) before progressively declining. It is theorized that the L* value is correlated with the samples’ moisture content and that the water that is extracted from the chicken meat during the heat treatment procedure sticks to the meat’s surface, raising the L* value. Following heating and cooking, the chicken meat’s a* value increased dramatically (*p* < 0.05), most likely as a result of myoglobin reacting with the meat’s remaining oxygen during the first heating stage and the meat’s increased redness value. The infiltration of brine broth during heating could be the cause. The penetration of brine broth during heating is likely the reason why the b* value of chicken flesh grew dramatically after cooking and stayed constant while stewing. It is evident from the data that the three cups of chicken meat showed greater color and more consistent variations.

### 3.3. The Analysis of Moisture Content

Preserving meat’s moisture content is crucial because yield and moisture content are the primary criteria used to assess the overall quality of meat products [30]. As shown in Figure 1D, four peaks were detected during the stewing process of the three-cup chicken: a smaller portion (T_2b_) [31], bound water (T_21_), fixed water (T_22_), and free water (T_23_) [32]. As illustrated in Figure 1D, the oil peak might have been the first vibrational relaxation peak to show up in this experiment. As the stewing time increased, the chicken meat’s water content dropped (Figure 1E), and all of the low-field NMR peaks shifted to the left, indicating a drop in both water mobility and water content [33]. As stewing time increased, the T_23_ peak area exhibited a pronounced increase, while the T_22_ peak area demonstrated a concomitant decrease. This inverse correlation indicates a progressive conversion of immobilized water into free water, attributable to heat-induced protein denaturation and the disruption of myofibrillar structures, which compromises the matrix’s capacity to retain water. In contrast, the T_21_ peak area remained relatively stable, confirming that the water most tightly associated with protein molecules was largely unaffected by the thermal process. This could be as a result of heat-induced protein denaturation, which also lessens meat’s softness by encouraging muscle fiber contraction in both longitudinal and transverse directions [34].

### 3.4. The Analysis of E-Nose

**Figure 2:**
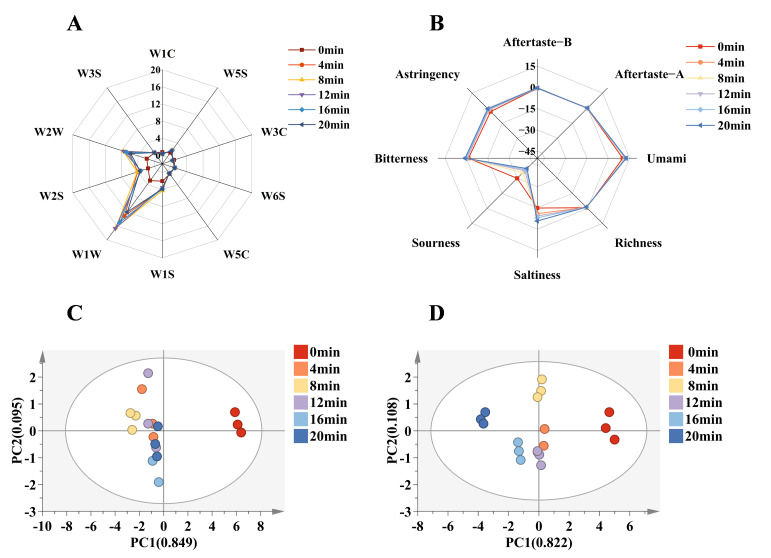
E-nose and E-tongue results of three-cup chicken with different stewing times (**A**) Radar chart of electronic nose response, (**B**) Radar chart of electronic tongue response, (**C**) Principal component diagram of electronic nose, (**D**) Principal component diagram of electronic tongue.

With a response value greater than 1, the W1W (sulfides, terpenes), W2W (aromatic components and organosulfides), W5S (nitrogen oxides), W1S (methane, various compounds), and W2S (alcohols, aldehydes, and ketones) receptors displayed a stronger response, as seen in the Figure 2A. This suggests that compounds like aromatic chemicals, sulfides, nitrogen oxides, methane, alcohols, aldehydes, and ketones had the biggest influence on the three-cup chicken’s odor. Overall flavor was provided by the presence of nitrogen oxides and sulfides [35], and the e-nose signal of the three-cup stewed chicken samples differed markedly from that of raw meat(*p* < 0.05). There were no appreciable variations across the chicken samples, although they did react to the sensors W3S (long chain alkanes) and W6S (hydrides) with values near 1. On the contrary, the response of the three cups of chicken samples to the sensor W3C (ammonia, aromatic components), W5C (short-chain alkanes, aromatic components), and W1C (aromatic components) is smaller, less than 1. Despite having the greatest response values, the electronic nose sensors W1W (sensitive to inorganic sulfur compounds) and W2W (sensitive to organic sulfur compounds) displayed a tendency to first increase and then decrease, which is consistent with the GC-MS results. It can be attributed to the initial production of a significant amount of volatile sulfur-containing compounds early in the stewing process. As stewing progressed, these compounds likely underwent further reactions, forming non-volatile macromolecular substances.

To further investigate flavor differences among the samples, PCA was conducted, as seen in Figure 2C. According to the PCA plot, both PC1 and PC2’s cumulative variance contributions exceeded 90%, suggesting that these two principal components may more accurately represent the samples’ overall flavor distribution and thus dimensionality reduction may result in less information loss. While the samples formed distinct clusters along PC2, indicating some variation in specific flavor attributes, their close proximity along PC1 suggests a fundamental similarity in their overall flavor profiles, particularly in the dominant taste components. When the stewing period exceeded 12 min, there were fewer variances between the three-cup chicken samples. The electronic nose was able to determine the samples’ general flavor profile, but it was unable to identify particular flavor chemicals, which require additional GC-MS analysis.

### 3.5. The Analysis of the Electronic Tongue

A key factor in assessing the quality of food is taste [36]. To analyze, identify, and evaluate the tested sample, the electronic tongue mimics the human tongue. Multivariate statistical techniques are then used to process the data, which rapidly reflects the sample’s overall quality information and enables sample identification and classification. As shown in Figure 2B, its taste response values were primarily concentrated in salty and umami taste, while the response values of sourness taste were lower, which was consistent with the traditional taste of three-cup chicken with salty and umami taste. The trend of taste response values detected by the electronic tongue sensor for three-cup chicken with varying stewing times was nearly the same. The occurrence of the *Maillard* reaction brought on by high-temperature stewing and the subsequent production of macromolecules may be the source of the minor increase in bitter value during stewing. During the stewing process, the electronic tongue’s umami-tasting response value peaked and trended upward in combination with the free amino acid data. The dissolution of organic acids during stewing and the consumption of the reaction may be the cause of the three cups of chicken’s decreased sourness value after stewing.

Figure 2D displayed the results of the PCA analysis of the taste information data obtained from the chicken sample. The sample taste information obtained by principal components 1 and 2 can essentially reflect the overall taste information of the original sample data, as evidenced by the contribution rate of principal components 1 and 2 reaching 90%. The three-cup chicken sample taste varied depending on how long it was stewed. Generally speaking, the stewing method greatly enhanced the three-cup chicken’s umami and salty qualities while decreasing its sour taste.

### 3.6. The Analysis of the FAA

The amino acids measured included bitter amino acids (His, Met, Tyr, Lys, Ile, Leu, Phe, Val), sweet amino acids (Ser, Gly, Thr, Ala, Pro), and umami amino acids (Asp, Glu) [20]. Huan et al. [37] used PLSR analysis to study the correlation between non-volatile compounds and sensory properties and concluded that FAA is essential for umami, while FAA such as Gly, Ala, Lys and Ser contribute to sweetness, and Glu and Asp are related to umami.

The free amino acid contents in three-cup chicken with different stewing times are shown in Table 2. Among them, glutamic acid content was the highest and much higher than the threshold value, which contributed the most to the umami flavor taste of three cups of chicken, and the glutamic acid content showed a gradual increase in the process of stewing, which was consistent with the trend of the electronic tongue’s umami flavor value. In this study, the increase of free glutamic acid during stewing is the result of the combined effect of endogenous release and exogenous addition: through hydrothermal hydrolysis, the heat generated during stewing breaks down proteins into the amino acids they constitute. This process is called hot hydrolysis, which releases the naturally occurring bound glutamic acid in these proteins, significantly increasing its free and umami form in the soup [38]. Meanwhile, during the stewing process of three-cup chicken, soy sauce is added. Soy sauce is a well-known source rich in free glutamic acid (usually in the form of sodium glutamate). Adding soy sauce directly to the stewed soup will introduce a large amount of umami compounds from the outside, thereby immediately and directly increasing the total amount of glutamic acid in the three-cup chicken. In conclusion, the increase in glutamic acid is attributed not only to the endogenous production resulting from protein breakdown but also to the direct contribution brought by the addition of soy sauce. During the stewing process, the content of various amino acids in the three-cup chicken shows an upward trend. This might be due to the fact that as the thermal processing time extends, the large protein molecules in the chicken are decomposed into small free amino acids and short peptides, which makes the three-cup chicken rich in flavor and more umami after stewing [39]. The FAA content in traditional braised pork also increases during the stewing process [40].

The lack of a significant difference in the E-tongue’s umami reading, despite the measured increase in glutamic acid, is because taste perception is non-linear. Once the glutamic acid content surpasses a certain threshold, further increases produce diminishing returns in perceived intensity. Furthermore, the overall umami taste is a complex result of interactions with other taste components in the matrix, which may have masked the subtle change in glutamate, resulting in a stable sensor response across samples.

### 3.7. The Analysis of GC-IMS

A total of 61 volatile components, including 15 ketones, 10 aldehydes, 10 alcohols, 7 aromatics, 6 esters, 3 ethers, 3 hydrocarbons, 2 acids, and 5 other compounds, were identified by GC-IMS analysis. With OPLS-DA, an analysis of variance was conducted. The OPLS-DA model fit the data satisfactorily, as indicated by R^2^X and R^2^Y values of 0.985 and 0.996, respectively. 16 compounds in all with VIP values greater than one were chosen as possible candidates for group-to-group aroma compound differentiation. In the meantime, the primary chemical classes identified by GC-IMS were determined to be aldehydes and ketones. The letter “D” stands for the 18 dimers that were generated in the IMS drift tubes.

By using GC-IMS and GC-MS, five volatile flavor compounds were both identified, including acetophenone, p-cymene, 1-octen-3-ol, dimethyl trisulfide, and methional. The volatile aroma compounds detected by GC-IMS were more short-chained volatile aroma substances than those detected by GC-MS, including 2-propanone, 3-pentanone, n-pentanal, and ethyl acetate, with most of them being C_3_-C_10_ molecules.

**Figure 3:**
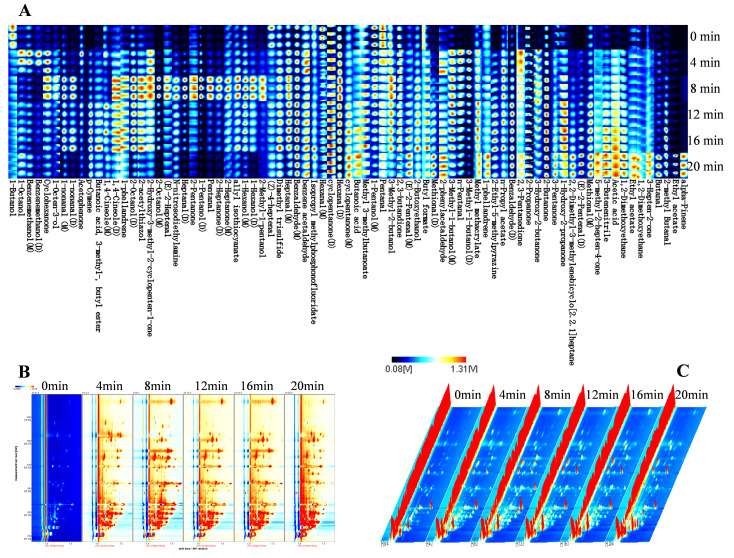
Volatile compounds of Three-cup chicken with different stewing times identified by GC-IMS. (**A**) The Gallery plot, (**B**) the comparison results of 2D topographic plots, (**C**) the 3D topographic plot. “M” and “D” denote monomer and dimer, respectively.

Combined with the fingerprint (Figure 3A), each point on the graph represents the volatile components in the sample, reflecting the color intensity of the concentration. To understand differences in the VOCs of the three-cup chicken at different stewing times, 2D and 3D topographies were created based on the signal intensity of each compound (Figure 3B,C). The graph indicates that the raw meat contains fewer flavoring compounds, and the content of flavor substances in the three cups of chicken changes during the braising process; benzaldehyde, 2-ethyl-5-methylpyrazine, and p-cymene gradually increase during the braising process; and ethyl acetate and alpha-pinene appear in the braising process at 20 min. During the stewing process, the contents of hexanal, methional, and phenylacetaldehyde gradually increased, while the content of 2-acetylthiazole showed little change. Hexanal has also been found to have a high odor activity in boiled chicken and Chinese Yellow-Feather chicken soup. 2-acetylthiazole is produced by the interaction between sugars and sulfur-containing AAs (cysteine or cystine) or peptides (GSH) and plays an important role in the formation of the flavor profile of chicken meat. As we know, methional, derived from Met, produces a cooked potato aroma, whereas phenylacetaldehyde from Phe offers a honey-like aroma.

### 3.8. The Analysis of GC-MS

**Figure 4:**
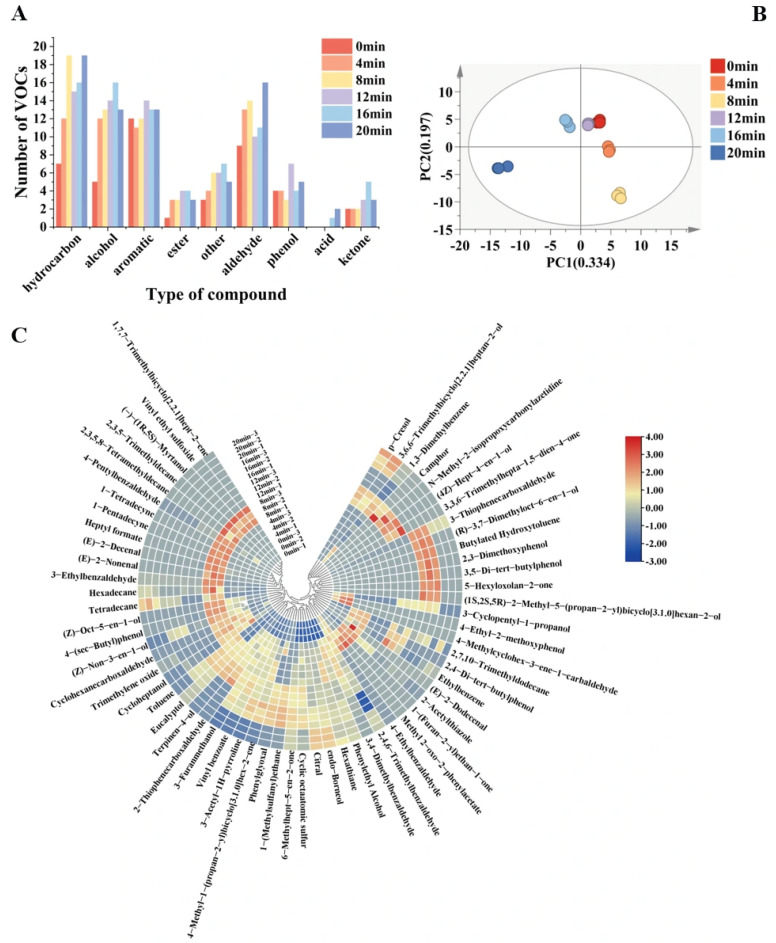
Analysis of VOCs in Three-cup chicken Clustering heat maps of VOCs at different stewing times identified by GC-MS (**A**) Types of VOCs found in chicken meat, (**B**) Heatmap of volatile compounds with VIP > 1, (**C**) OPLS-DA score plot.

The number of volatiles gradually increased during the stewing process, and a total of 29 hydrocarbons, 25 aldehydes, 23 alcohols, 20 aromatics, 14 ketones, 8 esters, 8 phenols, 2 acids were identified, and 8 others, for a total of 137 flavor compounds (Table S2); with hydrocarbons, aldehydes, alcohols, and ketones showing the most pronounced growth(Figure 4A). Key VOCs were identified based on odour activity value (OAV) >1, and Table 3 displays the findings. All samples included a total of 25 important VOCs, the majority of which were aromatics with aldehydes. Additionally, during the stewing process, the samples’ VOC levels underwent considerable variations, which resulted in modifications to their fragrance characteristics.

In order to explore the potential relationship between different stewing times and their effects on the volatile compound profiles of the three-cup chicken samples, OPLS-DA analysis was performed. This analysis assessed the correlation between different stewing times and the significant volatile compounds identified by GC-MS. Based on OPLS-DA analysis, the differences in flavor compounds in the addition of three cups of chicken with different stewing times were further explored. As shown in Figure 4C, the different stewing times of three-cup chickens showed good separation in the OPLA-DA score plots. There were 63 volatile chemicals found to have VIP values higher than 1. To give a clearer and more succinct visual representation of the changes in volatile components during the braising process of Three-Cup Chicken, a heatmap was created based on the concentrations of these VIP > 1 volatile compounds (as seen in Figure 4B). The OPLS-DA model’s RY^2^ and Q^2^ were 0.993 and 0.977 > 0.4, respectively, demonstrating the model’s stability and dependability. As a result, three-cup chicken with varying stewing periods can have its volatile taste compounds screened using the OPLS-DA model based on GC-MS data. In this study, the volatile constituents with VIP value >1 and OAV value >1 indicated significant differences in three-cup chickens with different stewing times and contributed significantly to the flavor. A total of 10 volatile components were considered to be significantly different, and a total of 10 key flavor substances were obtained, including 5-Hexyloxolan-2-one, 2-acetylthiazole, 1-(Methylsulfanyl)ethane, (E)-2-Decenal, (E)-2-Dodecenal, (E)-2-Nonenal, 4-Ethylbenzaldehyde, cyclohexanecarboxaldehyde, citral, and 6-Methylhept-5-en-2-one. The concentrations of these 10 key flavor substances showed distinct dynamic changes during stewing: 2-acetylthiazole, (E)-2-Decenal, citral, and 6-Methylhept-5-en-2-one exhibited increasing trends with prolonged stewing time, suggesting their continuous formation through thermal reactions. In contrast, 4-Ethylbenzaldehyde and cyclohexanecarboxaldehyde decreased progressively, possibly due to their degradation or transformation under extended heating. Notably, 5-Hexyloxolan-2-one, 1-(Methylsulfanyl)ethane, (E)-2-Dodecenal, and (E)-2-Nonenal appeared and subsequently disappeared during the stewing process, indicating their transient nature as intermediate reaction products.

Due to its low odor threshold, aldehydes play an important role in the characteristic flavor, and 25 aldehydes were detected in the three-cup chicken samples. Among them, the ones that contributed more to the aroma of the three-cup chicken during stewing were (E, Z)-2,4-ecadienal (with fat, green aroma), (E)-2-Decenal (waxy fat, earthy, green, cilantro, mushroom aldehydes); (E)-2-Dodecenal (citrus flavors); (E)-2-Nonenal (fat cucumber aldehydes citrus odors); 4-Ethylbenzaldehyde (almond bitter almond sweet anise cherry); cyclohexanecarboxaldehyde (strong pungent odor), citral (lemon, peppermint), and others. In addition, (E)-2-octenal, (E)-2-Decenal, (Z)-2-nonenal, (E, E)-2,4-decadienal, and (E)-2-hexenal have been reported to be formed by the oxidation of unsaturated fatty acids or by the thermo-oxidative decomposition of lipids in foods. These results are consistent with those previously reported for cooked chicken products [42].

*Maillard* reactions and the thermal oxidation and degradation of unsaturated fatty acids culminate in the formation of ketones. They frequently create fragrant scents with floral, creamy, and fruity aroma qualities, and they have a low threshold for flavor change. 6-Methyl-5-hepten-2-one, which has a fruity and fresh crisp aroma, was not found in raw beef. During the braising process, its concentration progressively rose and subsequently somewhat dropped. The only notable volatile ingredient in the preparation of braised meatballs in brown sauce is 6-methyl-5-hepten-2-one [43].

The oxidation of polyunsaturated fatty acids produces 1-octen-3-ol, one of the alcohols, which smells of mushrooms and greenery. While terpinen-4-ol and eucalyptol, two other alcohols that were primarily produced from tastes, were found in the samples, eucalyptol gave the sample a distinctive cooling scent [44].

Since hydrocarbons are mainly odorless or have a subtle odor and have a high odor threshold, they don’t directly affect the flavor of three-cup chicken. Lipids can degrade oxidatively to form aliphatic hydrocarbons, while free amino acids with aromatic groups can oxidize to produce aromatic hydrocarbons [12].

The reaction of fat-oxidized alcohol intermediates yields the ester molecules [45]. After 16 min of stewing, 5-Hexyloxolan-2-one was found in the sample and had a faintly fruity flavor. Moreno-Horn et al. [46] were the first to notice that ó-decalactone (5-hexyl-dihydro-2(3H)furanone, 1c) was naturally formed when fungi broke down ricinoleic acid. Oxygenated fatty acids were generally identified as the origins of yeast γ- and δ-lactones. In the process of making beer, linoleic acid is saccharified by enzymatic or auto-oxidation, resulting in hydroperoxides. These hydroperoxides are then transformed into 4-hydroxynonanoic acid, 2(3H)-furanone, and dihydro-5-pentyl-(γ-nonanolactone) [47].

Dimethyl trisulfide is produced mainly by Strecker degradation of methionine and is an important contributor to meat flavor [48]. This may be responsible for the enhancement of meat flavor with longer braising time.

Long-term heating conditions produce a range of reactive carbonyl compounds from free amino acids and reducing sugars through *Maillard* reactions and Strecker degradation, which gives meat its distinct flavor. These substances are essential for the synthesis of heterocyclic compounds [49]. The primary flavoring compounds created when meat is heated are furans, which are byproducts of the *Maillard* reaction. For instance, linoleic acid oxidizes 2-pentylfuran, a noncarboxylated molecule that is generated from linoleic acid and other n-6 fatty acids. It has a vegetal aroma and a relatively low threshold. Linoleic acid does this by degrading methyl linoleate hydroperoxide. The most significant class of meat flavoring chemicals is thiophene. These flavoring chemicals, which are necessary in meat products, are found in large concentrations in 2-propylthiophene, 2-pentylthiophene, and 2-heptylthiophene. The samples contained 3-methylthiophene, 3-thiophenecarboxaldehyde, 2-thiophenecarboxaldehyde, and 5-methyl-2-thiophenecarboxaldehyde. An alkyl chain thiazole. A breakdown product in the morphological route involving fatty aldehydes could be the cause of this [50]. 2-Acetylthiazole was detected in the samples with an aroma similar to that of nuts, rice, and popcorn, which showed a tendency to increase and then decrease during stewing.

### 3.9. Correlation Analysis

**Figure 5:**
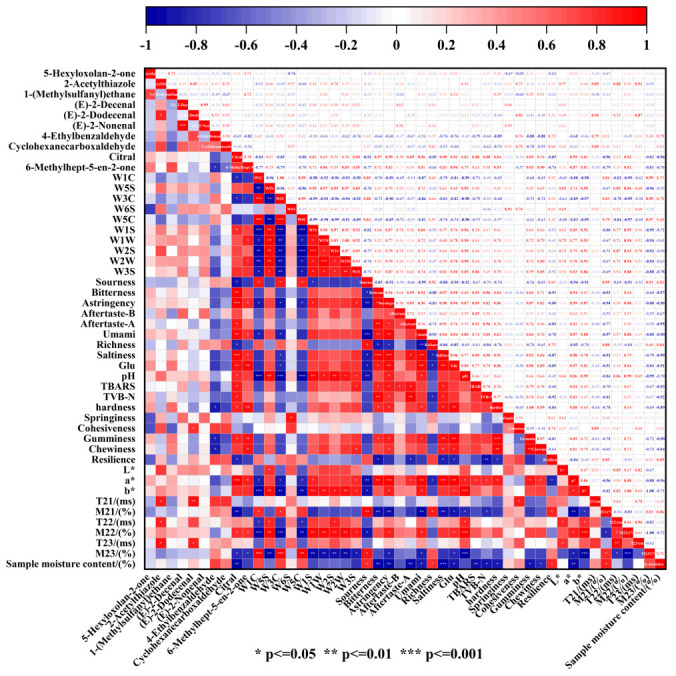
Heatmap of correlation between quality and flavor indicators of three-cup chicken.

A correlation study was built utilizing certain quality and flavor indicators to acquire a better understanding of the rule of flavor change during the stewing process of three-cup chicken. The quality and flavor of three-cup chicken are strongly correlated with each other throughout the cooking process, as shown in Figure 5. The significant positive correlation between the T_2_ relaxation time and key volatiles like 2-acetylthiazole and (E)-2-dodecenal suggests that increased water mobility facilitates the release and formation of *Maillard* reaction and lipid oxidation-derived flavor compounds. This is mechanistically explained by water loss concentrating reactants and providing a medium for their interaction. There was a substantial correlation between the proportion of relaxation signal components (M_2_) and redness and yellowness. Water loss hurried up the *Maillard* reaction in the three-cup chicken, although moisture content and water activity decreased. The strong positive correlation between glutamate content and the e-tongue’s umami value directly confirms that the dramatic increase in this free amino acid, derived from protein hydrolysis and soy sauce addition, is the fundamental contributor to the savory taste of the dish. Significant positive correlations were found between 6-Methylhept-5-en-2-one, citral, and TBARS and TVB-N values. This suggests that during the stewing process, protein oxidation and breakdown can encourage the formation of volatile chemicals, particularly those with a pleasant odor. Furthermore, lipid oxidation is the primary process that produces the volatile chemicals aldehydes and allyl aldehydes, it also provide a crucial insight: controlled protein and lipid oxidation during stewing is not merely a spoilage indicator but a key driver for the formation of desirable aromatic compounds, particularly aldehydes and ketones. Based on the correlation, it is evident that the degree of lipid oxidation during the stewing process is positively connected with the degree of protein oxidation.

The development of pleasant volatile flavor compounds and the enhancement of taste appearance brought about by the *Maillard* reaction, myofibrillar protein aggregation, protein and lipid oxidation, and water loss were the primary causes of the three-cup chicken’s quality during stewing.

## 4. Conclusions

This study systematically characterized the physicochemical and flavor dynamics of Three-Cup Chicken during its characteristic stewing process. As stewing time increased, the product exhibited intensified lipid oxidation, accompanied by rising hardness, redness (a*), and yellowness (b*). Concurrently, moisture content declined, reflecting a shift in water state characterized by an increased proportion of immobilized and bound water at the expense of free water. Flavor profiles, as determined by electronic nose and tongue, underwent substantial changes throughout the stewing process. GC-MS and GC-IMS analyses identified 137 volatile compounds, from which 10 key odorants—predominantly aldehydes—were screened based on variable VIP > 1 and OAV > 1. Most of these compounds exhibited an initial increase followed by a decrease during heating, suggesting thermally driven generation and subsequent degradation. These findings explicitly validate Three-Cup Chicken’s characteristic sensory profile: rich color, aromatic complexity, and dominant salty-umami taste. Based on the comprehensive experimental results, a stewing time of 20 min is recommended for achieving the most favorable balance of taste, texture, and overall quality in the prepared product. This study delivers a robust scientific basis for the standardized and industrialized production of Three-Cup Chicken, directly responding to the growing demand for high-quality pre-prepared traditional foods. Future research should focus on clarifying the precise reaction pathways and metabolic interactions that underpin the distinctive flavor development in three-cup chicken.

## Figures and Tables

**Table 1:** Effects of different stewing times on the texture and color of the three-cup chicken.

Stewing Time	0 min	4 min	8 min	12 min	16 min	20 min
hardness (Kg)	0.38 ± 0.03 ^d^	1.34 ± 0.16 ^c^	2.71 ± 0.17 ^b^	4.05 ± 0.12 ^a^	4.10 ± 0.54 ^a^	4.30 ± 0.11 ^a^
Springiness (mm)	0.87 ± 0.04 ^bcd^	0.91 ± 0.13 ^abc^	1.01 ± 0.05 ^a^	0.99 ± 0.03 ^ab^	0.76 ± 0.01 ^d^	0.82 ± 0.07 ^bcd^
Cohesiveness	0.67 ± 0.04 ^b^	0.73 ± 0.03 ^a^	0.67 ± 0.04 ^b^	0.66 ± 0.02 ^bc^	0.61 ± 0.01 ^c^	0.65 ± 0.02 ^bc^
Gumminess (Kg)	0.25 ± 0.01 ^d^	0.98 ± 0.11 ^c^	1.81 ± 0.09 ^b^	2.68 ± 0.14 ^a^	2.50 ± 0.35 ^a^	2.80 ± 0.16 ^a^
Chewiness (Kg)	0.22 ± 0.02 ^e^	0.88 ± 0.08 ^d^	1.84 ± 0.06 ^c^	2.66 ± 0.16 ^a^	1.90 ± 0.24 ^c^	2.29 ± 0.25 ^b^
Resilience	0.74 ± 0.05 ^a^	0.67 ± 0.05 ^ab^	0.56 ± 0.12 ^c^	0.58 ± 0.01 ^bc^	0.27 ± 0.01 ^d^	0.29 ± 0.03 ^d^
L*	48.02 ± 3.8 ^c^	67.42 ± 1.65 ^a^	66.04 ± 1.21 ^a^	64.57 ± 2.66 ^a^	57.92 ± 2.08 ^b^	49.35 ± 3.69 ^c^
a*	−1.73 ± 0.09 ^c^	6.09 ± 1.64 ^b^	7.18 ± 0.9 ^b^	7.25 ± 0.82 ^b^	9.91 ± 0.88 ^a^	11.34 ± 1.08 ^a^
b*	1.39 ± 0.48 ^b^	13.13 ± 0.26 ^a^	11.98 ± 1.53 ^a^	13.32 ± 0.97 ^a^	12.18 ± 0.57 ^a^	12.09 ± 0.69 ^a^

Notes: Different letters within the same row indicate significant differences (*p* < 0.05).

**Table 2:** Analysis of free amino acid content of three-cup chicken with different stewing times.

Free Amino Acids	Taste Threshold (mg/100 g)	Concentrations (mg/100 g)					
		0 min	4 min	8 min	12 min	16 min	20 min
Aspartic acid (Asp)	100	4.56 ± 0.13 ^d^	22.5 ± 0.33 ^a^	16.01 ± 0.21 ^c^	16.37 ± 0.5 ^c^	20.47 ± 1.24 ^b^	16.84 ± 0.48 ^c^
Glutamic acid (Glu)	30	45.51 ± 0.95 ^f^	266.86 ± 3.96 ^e^	314.69 ± 5.88 ^d^	411.79 ± 10.82 ^c^	468.23 ± 26.79 ^b^	575.56 ± 15.04 ^a^
umani taste amino (Uaa)		68.72 ± 1.48 ^f^	396.57 ± 5.88 ^e^	451.82 ± 8.3 ^d^	584.36 ± 15.44 ^c^	667.24 ± 38.27 ^b^	807.68 ± 21.17 ^a^
Threonine (Thr)	260	20.79 ± 0.39 ^d^	52.91 ± 0.68 ^b^	46.07 ± 0.93 ^c^	46.46 ± 1.63 ^c^	59.09 ± 3.59 ^a^	50.73 ± 1.43 ^b^
Serine (Ser)	150	13.27 ± 0.2 ^d^	36.85 ± 0.39 ^ab^	30.95 ± 0.55 ^c^	31.89 ± 0.96 ^c^	37.68 ± 2.09 ^a^	35.32 ± 1.01 ^b^
Glycine (Gly)	60	18.67 ± 0.17 ^e^	30.18 ± 0.65 ^b^	25.92 ± 0.37 ^d^	27.75 ± 0.57 ^c^	32.41 ± 1.74 ^a^	29.35 ± 0.82 ^b^
Alanine (Ala)	130	19.56 ± 0.27 ^d^	56.24 ± 0.73 ^b^	50.67 ± 0.8 ^c^	50.73 ± 1.34 ^c^	60.94 ± 3.44 ^a^	56.56 ± 1.48 ^b^
Proline (Pro)	300	5.5 ± 0.35 ^d^	23.02 ± 0.78 ^b^	17.46 ± 0.09 ^c^	18.98 ± 0.98 ^c^	24.08 ± 1.23 ^b^	26.79 ± 1.13 ^a^
sweet taste amino acids (Saa)		148.18 ± 1.91 ^d^	366.89 ± 3.62 ^b^	315.69 ± 4.75 ^c^	325.49 ± 9.55 ^c^	392.66 ± 21.8 ^a^	366.99 ± 10.49 ^b^
Valine (Val)	40	3.48 ± 0.19 ^e^	26.32 ± 0.39 ^b^	22.1 ± 0.37 ^d^	22.95 ± 0.53 ^d^	29.05 ± 1.71 ^a^	24.85 ± 0.55 ^c^
Methionine (Met)	30	6.4 ± 0.21 ^c^	14.48 ± 0.3 ^a^	12.13 ± 0.19 ^b^	12.49 ± 0.55 ^b^	15.2 ± 0.67 ^a^	12.49 ± 0.64 ^b^
Isoleucine (Ile)	90	10.62 ± 0.24 ^e^	20.48 ± 0.35 ^bc^	17.94 ± 0.31 ^d^	19.5 ± 0.39 ^c^	23.73 ± 1.34 ^a^	21.51 ± 0.43 ^b^
Leucine (Leu)	190	11.16 ± 0.23 ^e^	35.91 ± 1.18 ^c^	32.25 ± 0.58 ^d^	34.39 ± 0.64 ^c^	41.66 ± 2.29 ^a^	38.03 ± 0.68 ^b^
Tyrosine (Tyr)		0 ± 0 ^e^	23.96 ± 0.98 ^a^	22.72 ± 0.4 ^b^	19.94 ± 0.21 ^d^	21.34 ± 0.82 ^c^	23.64 ± 0.21 ^ab^
Phenylalanine (Phe)	90	29.94 ± 0.44 ^d^	39.35 ± 0.26 ^b^	38.51 ± 0.73 ^b^	36.65 ± 0.66 ^c^	37.85 ± 1.72 ^bc^	49.08 ± 1.01 ^a^
Histidine (His)	20	1.79 ± 0.05 ^e^	8.31 ± 0.32 ^a^	5.64 ± 0.01 ^c^	5.52 ± 0.14 ^c^	7.43 ± 0.37 ^b^	5.03 ± 0.1 ^d^
Lysine (Lys)	50	11.77 ± 0.47 ^e^	32.34 ± 0.99 ^b^	22.84 ± 2.85 ^d^	22.31 ± 0.81 ^d^	37.33 ± 1.58 ^a^	29.59 ± 0.24 ^c^
bitter taste amino acids (Baa)		102.4 ± 1.57 ^d^	279.37 ± 2.95 ^b^	240.73 ± 3.86 ^c^	242.1 ± 4.71 ^c^	299.7 ± 14.95 ^a^	282.44 ± 3.67 ^b^
total amino acids (Taa)		319.3 ± 4.42 ^e^	1042.83 ± 12.08 ^d^	1008.24 ± 15.58 ^d^	1151.95 ± 29.3 ^c^	1359.61 ± 74.97 ^b^	1457.11 ± 34.71 ^a^

Notes: Different letters within the same row indicate significant differences (*p* < 0.05). The taste threshold values of FAAs referred to the study of Xi et al. [41].

**Table 3:** Volatile compounds with OAV > 1 during the stewing process of three-cup chicken.

Substance Name	CAS	Odor Threshold (μg/kg)	Concentrations (μg/kg)					
			0 min	4 min	8 min	12 min	16 min	20 min
Styrene	100-42-5	65	86.35 ± 4.2	56.13 ± 7.96	50.71 ± 0.06	85.29 ± 2.42	49.5 ± 5.18	239.97 ± 11.06
1-Octen-3-ol	3391-86-4	1.5	93.78 ± 25.35	113.44 ± 22.65	163.7 ± 0.94	26.67 ± 0.84	19.78 ± 1.1	17.02 ± 1.5
Naphthalene	91-20-3	6	7.92 ± 1.25	12.39 ± 2.34	15.76 ± 0.71	10.6 ± 1.18	9.85 ± 1.77	19.52 ± 0.77
2-Methylnaphthalene	91-57-6	3	1.93 ± 0.48	2.74 ± 0.16	6.2 ± 0.53	2.36 ± 0.29	2.38 ± 0.22	6.61 ± 1.26
(2-Methylpropyl)benzene	538-93-2	0.8						4.39 ± 0
p-Cymene	99-87-6	5.01		33.87 ± 35.03	16.67 ± 0.55	13.62 ± 1.5	14.87 ± 0.63	16.92 ± 1.74
2-Butylpyridine	5058-19-5	1.8	5.2 ± 7.82	22.6 ± 15.43	13.78 ± 0.72	3.11 ± 1		
2-Pentylfuran	3777-69-3	5.8	41.71 ± 31.29	33.18 ± 12.92	58.44 ± 2.5	9.09 ± 7.73		
5-Hexyloxolan-2-one	706-14-9	1.1					6.14 ± 0.31	
2-Acetylthiazole	24295-03-2	3	3.89 ± 0.34	35.67 ± 1.31	22.26 ± 0.97	8.96 ± 0.86	11.71 ± 1.08	6.83 ± 0.76
1-(Methylsulfanyl)ethane	624-89-5	22				20.72 ± 1.33	25.52 ± 0.35	
Dimethyl trisulfide	3658-80-8	0.1				8.53 ± 3.99	11.62 ± 7.26	
Carbon disulfide	75-15-0	5			18.91 ± 16.32		132.73 ± 24.9	329.42 ± 75.79
(E)-2-Decenal	3913-81-3	17	5.7 ± 2.01	18.26 ± 5.29	120.05 ± 9.65	6.44 ± 4.07	6.43 ± 1.66	10.42 ± 0.38
(E)-2-Dodecenal	20407-84-5	1.4		26.84 ± 24.13				
(E)-2-Nonenal	18829-56-6	0.19			43.38 ± 8.79			
Methional	3268-49-3	0.45						23.95 ± 5.64
4-Ethylbenzaldehyde	4748-78-1	40	30.97 ± 1.52	38.75 ± 11.39				
Neral	106-26-3	53						190.5 ± 28.48
Cyclohexanecarboxaldehyde	2043-61-0	25		27.76 ± 5.19	28.62 ± 1.93			
Citral	5392-40-5	28		205.1 ± 21.66	240.31 ± 4.87	210.37 ± 5.97	295.17 ± 15.65	379.99 ± 8.63
(E, Z)-2,4-Decadienal	25152-83-4	0.04		40.86 ± 53.84	12.94 ± 1.2		3.15 ± 0.26	12.29 ± 4.05
5-Methyl-2-thiophenecarboxaldehyde	13679-70-4	1.75		1.38 ± 0.13	2.03 ± 0.19	1.45 ± 0.58	2.35 ± 0.25	5.16 ± 0.56
(E)-2-Octenal	2548-87-0	3	30.27 ± 7.19	61.81 ± 14.94	61.59 ± 2.61	11.66 ± 1.17	9.29 ± 0.55	
6-Methylhept-5-en-2-one	110-93-0	68		60.87 ± 6.18	98.67 ± 3.04	133.7 ± 5.02	148.11 ± 4.77	105.5 ± 1.23

Notes: Empty cells indicate that the compound was not detected.

## Data Availability

The original contributions presented in the study are included in the article/Supplementary Materials. Further inquiries can be directed to the corresponding author.

## Supplementary Materials

The following supporting information can be downloaded at: https://www.mdpi.com/article/10.3390/foods14223970/s1, Table S1: Standard sensor array for electronic nose PEN3, Table S2: The names, CAS numbers, molecular formulas, flavor thresholds, and relative contents of volatile substances during the stewing process of three-cup chicken.
