# Supplementary material for: Dynamics Evolution of Flavor and Quality Attributes in Three-Cup Chicken: Insights from Multi-Technical Analysis During Stewing
Source: Foods. 2025 Nov 19;14(22):3970. doi: 10.3390/foods14223970 (PMC12652035; doi:10.3390/foods14223970)
Supplement: Supplementary file 1 [file foods-14-03970-s001.zip › foods-3954588-supplementary.pdf]

Table S1 Standard sensor array for electronic nose PEN3

Sensor serial number	Sensor name	Function description
S_1	W1C	sensitive to aromatic compounds
S_2	W5S	particularly sensitive to nitrogen oxide compounds
S_3	W3C	sensitive to ammonia and aromatic compounds
S_4	W6S	sensitive to hydrogen
S_5	W5C	sensitive to olefin and aromatic compounds
S_6	W1S	sensitive to methane and other hydrocarbons
S_7	W1W	sensitive to sulfides compounds
S_8	W2S	sensitive to alcohols and aroma compounds
S_9	W2W	sensitive to aromatic and organic sulfide compounds
S_{10}	W3S	sensitive to olefin compounds

Table S2. The names, CAS numbers, molecular formulas, flavor thresholds, and relative contents of volatile substances during the stewing process of three-cup chicken.

Type of substance	Componds	CAS	molecular formula	Thresholds (μg/kg)	relative content（μg/kg)					
					0min	4min	8min	12min	16min	20min
Hydrocarbons	Spiro[2,4]hepta-4,6-diene	765-46-8	C7H8	ND	29.03±16.81	104.06±10.16	101.55±5.78	114.76±6.08	178.53±16.99	219.12±11.58
	Benzene, 1,3-dimethyl-	108-38-3	C8H10	1000	36.73±1.98	16.73±1.92	18.78±3.92	38.63±1.93	13.01±1.11	23.59±0.33
	Styrene	100-42-5	C8H8	65	86.35±4.2	56.13±7.96	50.71±0.06	85.29±2.42	49.5±5.18	239.97±11.06
	Dodecane, 4,6-dimethyl-	61141-72-8	C14H30	ND	5.42±2.78	5.17±4.29	11.07±4.19	6.46±1.77	7.25±0.8	9.87±0.66
	Hexadecane	544-76-3	C16H34	500	2.5±0.18	3.7±0.7	12±3.57	4.75±0.53	3.9±0.05	6.33±0.68
	Decane, 2,3,5,8-tetramethyl-	192823-15-7	C14H30	ND	ND	ND	5.61±2.24	ND	ND	ND

Bicyclo[2.2.1]hept-2-ene, 1,7,7-trimethyl-	464-17-5	C10H16	ND	ND	ND	8.08±1.59	ND	ND	ND
Decane, 2,3,5-trimethyl-	62238-11-3	C13H28	ND	ND	ND	0.95±0.21	ND	ND	ND
1-Tetradecyne	765-10-6	C14H26	ND	ND	ND	35.57±1.43	ND	ND	ND
1-Pentadecyne	765-13-9	C15H28	ND	ND	ND	7.96±0.39	ND	ND	ND
cis-Hept-4-enol	6191-71-5	C7H14O	ND	ND	ND	ND	17.23±1.39	ND	ND
Bicyclo[3.1.1]hept-2-ene, 3,6,6-trimethyl-	4889-83-2	C10H16	ND	ND	ND	ND	ND	5.34±1.23	46.34±4.78
Cyclobutane, 1,1,2,3,3-pentamethyl-	57905-86-9	C9H18	ND	ND	ND	ND	ND	ND	1.32±0.26
Camphene	79-92-5	C10H16	26000	ND	ND	ND	ND	ND	9.53±0.64
Tridecane	629-50-5	C13H28	42000	ND	ND	ND	ND	ND	9.74±6.57
1H-3a,7-Methanoazulene, 2,3,6,7,8,8a-hexahydro-1,4,9,9-tetramethyl-, (1.alpha.,3a.alpha.,7.alpha.,8a.beta.)-	560-32-7	C15H24	ND	ND	ND	ND	ND	ND	4.02±0.63
1,3-Cyclohexadiene, 5-(1,5-dimethyl-4-hexenyl)-2-methyl-, [S-(R*,S*)]-	495-60-3	C15H24	ND	ND	ND	ND	ND	ND	203.11±34.94
Tridecane, 6-methyl-	13287-21-3	C14H30	ND	ND	ND	ND	2.4±1.78	2.18±1.54	ND
Bicyclo[3.1.0]hex-2-ene, 4-methyl-1-(1-methylethyl)-	28634-89-1	C10H16	ND	ND	6.96±5.48	20.69±0.77	14.53±0.4	22.9±2.88	ND
1H-Benzocycloheptene, 2,4a,5,6,7,8,9,9a-octahydro-3,5,5-trimethyl-9-methylene-, (4aS-cis)-	3853-83-6	C15H24	ND	ND	ND	34.17±4.32	ND	ND	107.7±7.39
Trimethylene oxide	503-30-0	C3H6O	ND	ND	6.25±1.43	8.58±0.6	ND	ND	1.46±0.54
Dodecane, 2,7,10-trimethyl-	74645-98-0	C15H32	ND	ND	8.87±0.72	ND	5.61±3.24	4.44±1.62	ND
Tetradecane	629-59-4	C14H30	1000	ND	ND	40.93±12.21	ND	21.13±1.03	42.83±8.57
trans-Calamenene	73209-42-4	C15H22	ND	ND	ND	5.35±0.39	2.17±0.03	12.26±5.16	16.62±0.63
1,3-Cyclohexadiene, 1-methyl-4-(1-methylethyl)-	99-86-5	C10H16	80	ND	1.38±0.11	1.38±0.7	1.31±0.27	1.62±0.56	ND
Benzene, 1-(1,5-dimethyl-4-hexenyl)-4-methyl-	644-30-4	C15H22	ND	ND	45.4±3.74	223.85±7.01	29.83±1.04	90.71±19.63	593.91±77.23
Cyclohexene, 3-(1,5-dimethyl-4-hexenyl)-6-methylene-, [S-(R*,S*)]-	20307-83-9	C15H24	ND	ND	4.05±0.69	16.77±1.75	3.5±0.11	24.19±4.86	104.82±9.47
Undecane, 4,7-dimethyl-	17301-32-5	C13H28	ND	7.61±6.66	ND	3.03±1.94	3.04±1.43	4.64±2.09	8.62±2.27
1-Tridecyne	26186-02-7	C13H24	ND	4.34±1.74	10.81±5.29	ND	8.31±1.25	7.22±1.3	18.58±2.04

	Cyclopentanol, 2-methyl-	24070-77-7	C6H12O	ND	135.68±86.18	332.94±126.47	504.51±24.48	62.56±2.92	46.23±1.59	51.73±1
	1-Octen-3-ol	3391-86-4	C8H16O	1.5	93.78±25.35	113.44±22.65	163.7±0.94	26.67±0.84	19.78±1.1	17.02±1.5
	3-Nonen-1-ol, (Z)-	10340-23-5	C9H18O	ND	161.61±8.1	498.69±149.32	859.29±48.48	276.7±37.44	551.69±38.64	117.51±10.21
	(-)-cis-Myrtanol	51152-12-6	C10H18O	ND	ND	ND	4.51±1.25	ND	ND	ND
	Bicyclo[3.1.0]hexan-2-ol, 2-methyl-5-(1-methylethyl)-, (1.alpha.,2.alpha.,5.alpha.)-	17699-16-0	C10H18O	55000	ND	ND	ND	ND	20.44±2.43	ND
	Benzyl alcohol	100-51-6	C7H8O	2546.21	ND	ND	ND	ND	5.84±4.29	ND
	6-Octen-1-ol, 3,7-dimethyl-, (R)-	1117-61-9	C10H20O	40	ND	ND	ND	ND	2.8±0.27	ND
	1H-Imidazole-4-methanol	822-55-9	C4H6N2O	ND	ND	ND	ND	ND	ND	7.22±0.69
	Bicyclo[3.1.1]heptane-2-methanol, 6,6-dimethyl-, [1S-(1.alpha.,2.alpha.,5.alpha.)]-	53369-17-8	C10H18O	ND	ND	ND	ND	ND	ND	6.71±0.12
Alcohol	5-Octen-1-ol, (Z)-	64275-73-6	C8H16O	6	ND	176.98±32.29	393.42±3.98	ND	ND	ND
	2,6-Octadien-1-ol, 3,7-dimethyl-, (Z)-	106-25-2	C10H18O	53	ND	ND	ND	ND	ND	13.86±1.75
	2-Norpinanol, 3,6,6-trimethyl-	29548-09-2	C10H18O	ND	ND	ND	ND	6.91±0.91	ND	9.92±1.79
	3-Cyclopentyl-1-propanol	767-05-5	C8H16O	ND	136.71±35.23	ND	ND	105.92±4.36	112.35±8.63	ND
	Phenylglyoxal	1074-12-0	C8H6O2	ND	ND	14.69±5.2	ND	15.88±1.21	18.87±0.81	ND
	9-Octadecen-1-ol, (Z)-	143-28-2	C18H36O	ND	ND	ND	ND	13.62±5.04	24.55±12.63	22.49±1.82
	Terpinen-4-ol	562-74-3	C10H18O	1200	ND	10.19±2.25	12.75±0.81	16.74±0.69	ND	ND
	Cycloheptanol	502-41-0	C7H14O	4800	46.71±10.7	133.78±39.49	119.62±4.89	ND	24.27±16.56	ND
	Eucalyptol	470-82-6	C10H18O	1.1	ND	ND	113.06±2.16	126.94±4.97	86.94±2.71	69.86±0.37
	endo-Borneol	507-70-0	C10H18O	180	ND	72±8.78	70.52±0.96	83.32±3.18	96.42±3.43	125.87±4.53
	.alpha.-Terpineol	98-55-5	C10H18O	1200	ND	25.04±2.84	35.67±0.48	77.26±6.14	98.1±4.74	130.77±7.34
	3-Furanmethanol	4412-91-3	C5H6O2	ND	ND	7.43±0.53	7.02±1.18	7.94±0.62	8.09±0.92	ND
	Phenylethyl Alcohol	22258	C8H10O	ND	ND	125.93±14.52	98.18±3.93	80.93±3.37	103.18±2.68	93.78±4.16
	Bicyclo[3.1.1]hept-3-en-2-ol, 4,6,6-trimethyl-, [1S-(1.alpha.,2.beta.,5.alpha.)]-	18881-04-4	C10H16O	ND	ND	88.18±11.93	95.01±1.79	105.36±2.16	147.67±5.05	195.59±15.97
	Ethylbenzene	100-41-4	C8H10	2205.25	5.05±0.62	16.9±5.92	3.37±2.04	21.26±9.24	1.43±0.63	8.42±2.5
Aromatic	Naphthalene	91-20-3	C10H8	6	7.92±1.25	12.39±2.34	15.76±0.71	10.6±1.18	9.85±1.77	19.52±0.77
	Naphthalene, 2-methyl-	91-57-6	C11H10	3	1.93±0.48	2.74±0.16	6.2±0.53	2.36±0.29	2.38±0.22	6.61±1.26

	Mesitylene	108-67-8	C9H12	700	14.83±19.12	ND	ND	ND	ND	ND
	Benzene, 1-ethyl-4-methyl-	622-96-8	C9H12	600	ND	ND	ND	15.1±22.59	ND	ND
	Benzene, (2-methylpropyl)-	538-93-2	C10H14	0.8	ND	ND	ND	ND	ND	ND
	Indole	120-72-9	C8H7N	40	ND	ND	ND	ND	ND	1.15±0.75
	Benzene, 1,2,4-trimethyl-	95-63-6	C9H12	260	11.22±11.31	ND	ND	ND	ND	14.7±0.56
	Toluene	108-88-3	C7H8	527	ND	ND	10.52±1.01	11.5±0.48	7.72±0.24	ND
	Benzene, 1-(1,5-dimethylhexyl)-4-methyl-	1461-02-5	C15H24	ND	ND	ND	2.22±0.28	ND	4.42±2.74	10.55±1
	Benzene, 1-methyl-3-(1-methylethenyl)-	1124-20-5	C10H12	ND	ND	ND	ND	20.36±3.63	43.02±29.7	22.47±3.42
	p-Cymene	99-87-6	C10H14	5.01	ND	33.87±35.03	16.67±0.55	13.62±1.5	14.87±0.63	16.92±1.74
	Pyridine, 2-butyl-	5058-19-5	C9H13N	1.8	5.2±7.82	22.6±15.43	13.78±0.72	3.11±1	ND	ND
	Furan, 2-pentyl-	3777-69-3	C9H14O	5.8	41.71±31.29	33.18±12.92	58.44±2.5	9.09±7.73	ND	ND
	Benzothiazole	95-16-9	C7H5NS	80	1.75±0.63	ND	ND	5.63±0.82	10.78±12.96	7.94±1.47
	Thiophene, 3-methyl-	616-44-4	C5H6S	ND	ND	9.61±2.13	12.25±2.89	10.8±0.54	15.73±0.89	18.99±0.87
	Benzene, 1,2,3-trimethyl-	526-73-8	C9H12	ND	8.24±6.61	4.11±0.68	ND	4.66±1.1	2.68±2.16	5.12±1.04
	3-Butylisobenzofuran-1(3H)-one	6066-49-5	C12H14O2	10	1.97±1.01	3.59±0.8	7.56±0.2	ND	3.26±2.1	ND
	Naphthalene, 1-methyl-	90-12-0	C11H10	7.5	1.47±0.05	1.48±0.5	2.01±0.36	1.57±0.04	1.35±0.13	ND
	Phthalic anhydride	85-44-9	C8H4O3	320	1.5±0.26	4.07±0.26	4.5±0.03	3.55±0.09	3.08±0.28	ND
Esters	Vinyl benzoate	769-78-8	C9H8O2	ND	12.25±2.52	1199.65±60.85	1074.91±33.92	1446.83±80.79	1080.01±47.7	26.4±3.1
	Benzeneacetic acid, .alpha.-oxo-, methyl ester	15206-55-0	C9H8O3	ND	ND	11.6±4.67	ND	ND	ND	ND
	Formic acid, heptyl ester	112-23-2	C8H16O2	ND	ND	ND	29.18±1.43	ND	ND	ND
	N-Methyl-2-isopropoxycarbonylazetidine	51764-30-8	C8H15NO2	ND	ND	ND	ND	0.46±0.15	ND	ND
	2(3H)-Furanone, 5-hexyldihydro-	706-14-9	C10H18O2	1.1	ND	ND	ND	ND	6.14±0.31	ND
	n-Caproic acid vinyl ester	3050-69-9	C8H14O2	ND	ND	40.25±15.15	57.91±1.91	4.24±1.37	ND	ND
	Linalyl acetate	115-95-7	C12H20O2	1000	ND	ND	ND	6.62±0.81	10.05±0.79	8.57±1.05
	Methyl salicylate	119-36-8	C8H8O3	40	ND	ND	ND	ND	2.56±0.48	3.18±0.48
Aldehyde	Benzaldehyde, 3,4-dimethyl-	5973-71-7	C9H10O	ND	1.79±0.15	55.54±3.58	61.69±1.02	47.4±4.41	31.58±0.87	27.4±0.99
	Isophthalaldehyde	626-19-7	C8H6O2	ND	1.81±0.29	2.51±1.01	4.09±0.41	4.03±1.4	6.73±0.68	4.06±0.11
	2-Decenal, (E)-	3913-81-3	C10H18O	17	5.7±2.01	18.26±5.29	120.05±9.65	6.44±4.07	6.43±1.66	10.42±0.38

	3-Cyclohexene-1-carboxaldehyde, 4-methyl-	7560-64-7	C8H12O	ND	24.25±7.53	ND	ND	ND	ND	ND
	2-Dodecenal, (E)-	20407-84-5	C12H22O	1.4	ND	26.84±24.13	ND	ND	ND	ND
	2-Nonenal, (E)-	18829-56-6	C9H16O	0.19	ND	ND	43.38±8.79	ND	ND	ND
	1,4-Benzenedicarboxaldehyde	623-27-8	C8H6O2	ND	ND	ND	2.75±0.19	ND	ND	ND
	3-Furaldehyde	498-60-2	C5H4O2	ND	ND	ND	ND	ND	ND	8.87±1.62
	Methional	3268-49-3	C4H8OS	0.45	ND	ND	ND	ND	ND	23.95±5.64
	2-Furancarboxaldehyde, 5-methyl-	620-02-0	C6H6O2	1110	ND	ND	ND	ND	ND	4.9±0.05
	Benzaldehyde, 4-ethyl-	4748-78-1	C9H10O	40	30.97±1.52	38.75±11.39	ND	ND	ND	ND
	Neral	106-26-3	C10H16O	53	ND	ND	ND	ND	ND	190.5±28.48
	Benzeneacetaldehyde, .alpha.-ethylidene-	4411-89-6	C10H10O	ND	ND	ND	ND	ND	ND	11.73±0.13
	.alpha.-Campholenal	4501-58-0	C10H16O	ND	ND	ND	ND	ND	4.51±2.37	4.31±0.02
	Cyclohexanecarboxaldehyde	2043-61-0	C7H12O	25	ND	27.76±5.19	28.62±1.93	ND	ND	ND
	3-Thiophenecarboxaldehyde	498-62-4	C5H4OS	ND	ND	ND	ND	ND	8.03±0.23	3.33±0.16
	3,6-Octadienal, 3,7-dimethyl-	55722-59-3	C10H16O	ND	ND	ND	ND	ND	6.21±1.02	7.13±0.65
	Benzaldehyde, 4-pentyl-	6853-57-2	C12H16O	ND	2.86±2.42	1.66±1.05	11.56±0.41	1.96±0.22	ND	ND
	Citral	5392-40-5	C10H16O	28	ND	205.1±21.66	240.31±4.87	210.37±5.97	295.17±15.65	379.99±8.63
	2-Thiophenecarboxaldehyde	35857	C5H4OS	ND	ND	7.62±1.8	8.62±0.51	13.51±2.29	ND	ND
	2,4-Decadienal, (E,Z)-	25152-83-4	C10H16O	0.04	ND	40.86±53.84	12.94±1.2	ND	3.15±0.26	12.29±4.05
	Benzaldehyde, 3-ethyl-	34246-54-3	C9H10O	ND	ND	ND	109.42±1.87	15.44±1.33	17.11±0.52	19.67±0.73
	5-Methyl-2-thiophenecarboxaldehyde	13679-70-4	C6H6OS	1.75	ND	1.38±0.13	2.03±0.19	1.45±0.58	2.35±0.25	5.16±0.56
	Benzaldehyde, 2,4,6-trimethyl-	487-68-3	C10H12O	ND	2.07±0.33	2.62±0.17	1.87±0.24	1.91±0.11	ND	1.94±0.09
	2-Octenal, (E)-	2548-87-0	C8H14O	3	30.27±7.19	61.81±14.94	61.59±2.61	11.66±1.17	9.29±0.55	ND
Phenol	Phenol, 4-(1-methylpropyl)-	99-71-8	C10H14O	ND	6.42±0.95	16.58±3.44	37.2±0.09	6.7±1.03	8.79±0.18	7.9±0.38
	2,3-Dimethoxyphenol	5150-42-5	C8H10O3	ND	ND	ND	ND	ND	0.75±0.12	ND
	Butylated Hydroxytoluene	128-37-0	C15H24O	1000	ND	ND	ND	ND	2.26±0.12	ND
	Phenol, 3,5-bis(1,1-dimethylethyl)-	1138-52-9	C14H22O	ND	ND	ND	ND	ND	19.99±3.11	ND
	Phenol, 2-(1,1-dimethylethyl)-	88-18-6	C10H14O	50	ND	ND	ND	ND	ND	2.19±1.9
	Phenol, 4-ethyl-2-methoxy-	2785-89-9	C9H12O2	89.25	ND	ND	0.76±0.15	ND	2.74±3.33	ND
	2,4-Di-tert-butylphenol	96-76-4	C14H22O	500	2.33±0.46	20.88±0.69	ND	17.8±1.01	ND	ND
	p-Cresol	106-44-5	C7H8O	10	ND	ND	ND	3.44±0.37	ND	3.46±0.81
Ketone	Acetophenone	98-86-2	C8H8O	65	1.56±1.86	ND	ND	ND	ND	ND
	Ethanone, 2-(formyloxy)-1-phenyl-	55153-12-3	C9H8O3	ND	0.66±0.18	ND	ND	ND	ND	ND

	Ethanone, 1-(2-furanyl)-	1192-62-7	C6H6O2	15025.2	ND	2.7±0.29	ND	ND	ND	ND
	Camphor	76-22-2	C10H16O	4600	ND	ND	ND	4.81±3.15	ND	ND
	1,5-Heptadien-4-one, 3,3,6-trimethyl-	546-49-6	C10H16O	ND	ND	ND	ND	1.58±0.68	ND	ND
	(+)-2-Bornanone	464-49-3	C10H16O	1360	ND	ND	ND	ND	ND	6.53±0.52
	Ethanone, 1-(1H-pyrrol-2-yl)-	1072-83-9	C6H7NO	58585.25	ND	ND	ND	ND	ND	74.46±1.11
	Ethanone, 1-(4-methylphenyl)-	122-00-9	C9H10O	21	4.01±5.43	ND	ND	2.85±1.05	ND	ND
	Isopropyl phenyl ketone	611-70-1	C10H12O	ND	ND	ND	ND	ND	ND	1.07±0.39
	Propiophenone, 2,2',4',6'-tetramethyl-	2040-22-4	C13H18O	ND	ND	ND	ND	0.8±0.08	2.23±2.22	ND
	1-Benzoxepin-2(3H)-one, octahydro-	4441-65-0	C10H16O2	ND	9.35±3.96	13.64±7.4	23.75±1.53	ND	ND	ND
	5-Hepten-2-one, 6-methyl-	110-93-0	C8H14O	68	ND	60.87±6.18	98.67±3.04	133.7±5.02	148.11±4.77	105.5±1.23
	4H-Pyran-4-one, 2,3-dihydro-3,5-dihydroxy-6-methyl-	28564-83-2	C6H8O4	35000	ND	ND	ND	6.55±7.57	10.39±4.66	40.47±6.05
	3-Acetyl-1H-pyrroline	1072-82-8	C6H7NO	ND	ND	32.85±2.03	34.65±0.99	36.64±1.11	49.05±3.42	ND
Acids	Benzoic acid	65-85-0	C7H6O2	1000	ND	ND	ND	ND	ND	1.57±0.26
	Sorbic Acid	110-44-1	C6H8O2	ND	ND	ND	ND	ND	75.56±77.83	70.62±17.72
Others	2-Acetylthiazole	24295-03-2	C5H5NOS	3	3.89±0.34	35.67±1.31	22.26±0.97	8.96±0.86	11.71±1.08	6.83±0.76
	Cyclic octaatomic sulfur	10544-50-0	S8	ND	3.12±0.38	8.07±0.85	7.63±0.73	9.53±0.4	9.29±0.85	6.91±0.36
	Hexathiane	13798-23-7	S6	ND	10.62±1.75	31.95±4.16	46.66±0.81	34.3±1.06	36.68±1.12	36.68±4.54
	Vinyl ethyl sulfoxide	32568-51-7	C4H8OS	ND	ND	ND	19.81±3.17	ND	ND	ND
	Ethane, (methylthio)-	624-89-5	C3H8S	22	ND	ND	ND	20.72±1.33	25.52±0.35	ND
	Dimethyl trisulfide	3658-80-8	C2H6S3	0.1	ND	ND	ND	8.53±3.99	11.62±7.26	ND
	Carbon disulfide	75-15-0	CS2	5	ND	ND	18.91±16.32	ND	132.73±24.9	329.42±75.79
	Dimethyl sulfone	67-71-0	C2H6O2S	ND	ND	4.38±1.29	2.98±1.22	3.02±0.62	6.51±1.44	22.08±4.5
